# Characterization of potential superspreader farms for bovine tuberculosis: A review

**DOI:** 10.1002/vms3.358

**Published:** 2020-09-16

**Authors:** Helen R. Fielding, Trevelyan J. McKinley, Richard J. Delahay, Matthew J. Silk, Robbie A. McDonald

**Affiliations:** ^1^ Environment and Sustainability Institute University of Exeter Penryn Cornwall UK; ^2^ College of Medicine and Health University of Exeter Exeter Devon UK; ^3^ National Wildlife Management Centre Animal and Plant Health Agency Stonehouse Gloucestershire UK; ^4^ Present address: The Royal (Dick) School of Veterinary Studies The Roslin Institute University of Edinburgh Midlothian UK

**Keywords:** bovine tuberculosis, cattle, heterogeneity, livestock movements, superspreader

## Abstract

**Background:**

Variation in host attributes that influence their contact rates and infectiousness can lead some individuals to make disproportionate contributions to the spread of infections. Understanding the roles of such ‘superspreaders’ can be crucial in deciding where to direct disease surveillance and controls to greatest effect. In the epidemiology of bovine tuberculosis (bTB) in Great Britain, it has been suggested that a minority of cattle farms or herds might make disproportionate contributions to the spread of *Mycobacterium bovis*, and hence might be considered ‘superspreader farms’.

**Objectives and Methods:**

We review the literature to identify the characteristics of farms that have the potential to contribute to exceptional values in the three main components of the farm reproductive number ‐ R_f_: contact rate, infectiousness and duration of infectiousness, and therefore might characterize potential superspreader farms for bovine tuberculosis in Great Britain.

**Results:**

Farms exhibit marked heterogeneity in contact rates arising from between‐farm trading of cattle. A minority of farms act as trading hubs that greatly augment connections within cattle trading networks. Herd infectiousness might be increased by high within‐herd transmission or the presence of supershedding individuals, or infectiousness might be prolonged due to undetected infections or by repeated local transmission, via wildlife or fomites.

**Conclusions:**

Targeting control methods on putative superspreader farms might yield disproportionate benefits in controlling endemic bovine tuberculosis in Great Britain. However, real‐time identification of any such farms, and integration of controls with industry practices, present analytical, operational and policy challenges.

## Graphical abstract

In the epidemiology of bovine tuberculosis (bTB) in Great Britain, a minority of ‘superspreader farms’ might make disproportionate contributions to the maintenance and spread of infection. A minority of farms act as trading hubs that greatly augment connections in trading networks, herd infectiousness might be increased by the presence of supershedding individuals, and herd infectiousness might be prolonged due to undetected infections or repeated local transmission, via wildlife or fomites. Targeting control methods on putative superspreader farms might yield disproportionate benefits in controlling endemic bovine tuberculosis in Great Britain.

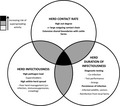

## 1 INTRODUCTION

In disease systems, superspreading individuals are defined by their tendency to generate disproportionately greater numbers of secondary infections when compared to other hosts (Lloyd‐Smith, Schreiber, Kopp, & Getz, 2005), thereby exerting a strong influence on disease dynamics. Epidemics in populations with superspreaders tend to be larger and more ‘explosive’ when outbreaks occur, but exhibit greater chances of infection dying out when the more numerous individuals with lower transmission rates are infected (Garske & Rhodes, 2008; Lloyd‐Smith et al., 2005). As the heterogeneity of the individual reproductive number (R_i_ – the number of secondary infections created from a single infected individual in a completely susceptible population) increases, there is wider variation in potential epidemic size, but the utility of the mean reproductive number (R_0_) reduces (Garske & Rhodes, 2008). Hence, epidemics have, for some diseases, been more effectively modelled by incorporating variation in R_i_ rather than assuming that the host population is homogeneous with regard to R_0_ (Lloyd‐Smith et al., 2005; Stein, 2011).

For livestock diseases, epidemics are often modelled using the farm, rather than the animal, as the epidemiological unit that acquires and spreads infection. The individual farm reproductive number, R_f_, is thus defined as the number of secondary farms infected by a primary infected farm (Mardones, Perez, Valdes‐Donoso, & Carpenter, 2011). R_f_ seems to show similar between‐individual variation as R_i_, with a minority of farms making a disproportionate contribution to secondary cases (VanderWaal et al., 2015; Woolhouse et al., 2005), apparently largely driven by variation in their trading behaviour (Woolhouse et al., 2005). R_f_ has been calculated in epidemics of foot and mouth disease (FMD) (Tildesley & Keeling, 2009), highly pathogenic avian influenza (HPAI) (te Beest, Hagenaars, Stegeman, Koopmans, & van Boven, 2011) and salmon infectious anaemia (Mardones et al., 2011). In FMD and HPAI models, reductions in epidemic size were achieved by targeting control measures on farms with higher R_f_, highlighting the benefits of identifying and targeting superspreader farms. While the impact of transmission heterogeneity has been evaluated for these highly‐transmissible diseases, the significance of R_f_ in endemic infections is less well‐understood. In contrast with incursions of exotic diseases, the ongoing nature of control measures for endemic infections, usually means that the population cannot be considered fully susceptible. Therefore, R_f_ might better be described as effective‐R_f_, though we refer to it here as R_f_ for brevity. Superspreader farms might be important in the dynamics of endemic infections (Brooks Pollock, Roberts, & Keeling, 2014), and long incubation and latency periods may allow undetected infection to spread further between farms (Dubé, Ribble, Kelton, & McNab, 2011). Brooks Pollock et al. (2014) constructed a dynamic, stochastic, spatial model of bovine tuberculosis (bTB) in Britain, using farm movements from the Cattle Tracing System (CTS) and bTB testing results to fit the model. They suggested that 10% of farms might be responsible for the majority of onward transmission to newly infected farms, implying that a disproportionate contribution from a minority of superspreader farms may play an important role in the maintenance and spread of this endemic infection. BTB is caused by infection with *Mycobacterium bovis* and is a major, ongoing problem for the British cattle industry (Allen, Skuce, & Byrne, 2018). Test and slaughter policies have previously reduced herd incidence (Department for Environment, Food & Rural Affairs, 2014), though since the 1980s increases in incidence have been accompanied by geographical spread from isolated hotspots to much of Wales and western England (Brunton et al., 2015). Control of bTB costs UK taxpayers more than £100 million annually and the financial and emotional impacts on farmers are substantial (Department for Environment Food & Rural Affairs, 2013). In 2018, 33,265 cattle (Animal & Plant Health Agency, 2019) and 32,601 European badgers (*Meles meles*) (Department for Environment, Food & Rural Affairs, 2018), which can constitute a wildlife reservoir of the infection (Godfray et al., 2013), were culled as part of bTB control measures in England. Cattle testing, predominantly using the Single Intradermal Cervical Comparative Tuberculin (SICCT) test, is currently mandatory on at least an annual basis in Wales and in the bTB High Risk Area (HRA) and Edge Area of England. Testing is required on a four‐yearly basis in the Low Risk Area (LRA) of England and in Scotland, apart from some exempt farms in Scotland, which is classed as Officially Tuberculosis Free (OTF). A positive reaction to the SICCT test, or lesions consistent with bTB found at slaughter, trigger movement restrictions on the affected farm. Initially, their OTF status is suspended (OTF‐S) and then withdrawn (OTF‐W) upon positive culture of *M. bovis*. Movement restrictions are lifted on the restoration of OTF status, typically after two consecutive herd tests with no positive results.

As bTB is a chronic, notifiable disease with mandatory control measures designed to find and eliminate disease, it might seem less important to consider superspreader farms in this context. Brooks Pollock et al. (2014) suggested that the majority of new bTB infections are caused firstly by cattle movements, and secondly through the local environment. Importantly, they also identified that a small number of farms are likely to create a disproportionate number of secondary cases, although they were not able to elaborate further on what might characterize these farms. Superspreader farms for bTB are likely to present differently to superspreaders of more highly transmissible diseases and could transmit infections over extended periods of time, in contrast with those that contribute to the steep epidemic curve of an outbreak. When characterizing superspreading farms, we should note that we are discussing not the commonalities of infection spread, but the circumstances that occur rarely, on a minority of important farms, and which might ‘evade’ control policies. Most studies of bTB epidemiology in cattle focus on herds that have experienced a bTB incident, i.e. where infection has been detected and the farm placed under restrictions. However, we focus on those herds that may be infected with bTB but where current infection has likely not been disclosed by testing and so farmers are able to sell cattle. The mechanisms by which farms might function as bTB superspreaders, may also be the very attributes that will make them difficult to identify.

**FIGURE 1:**
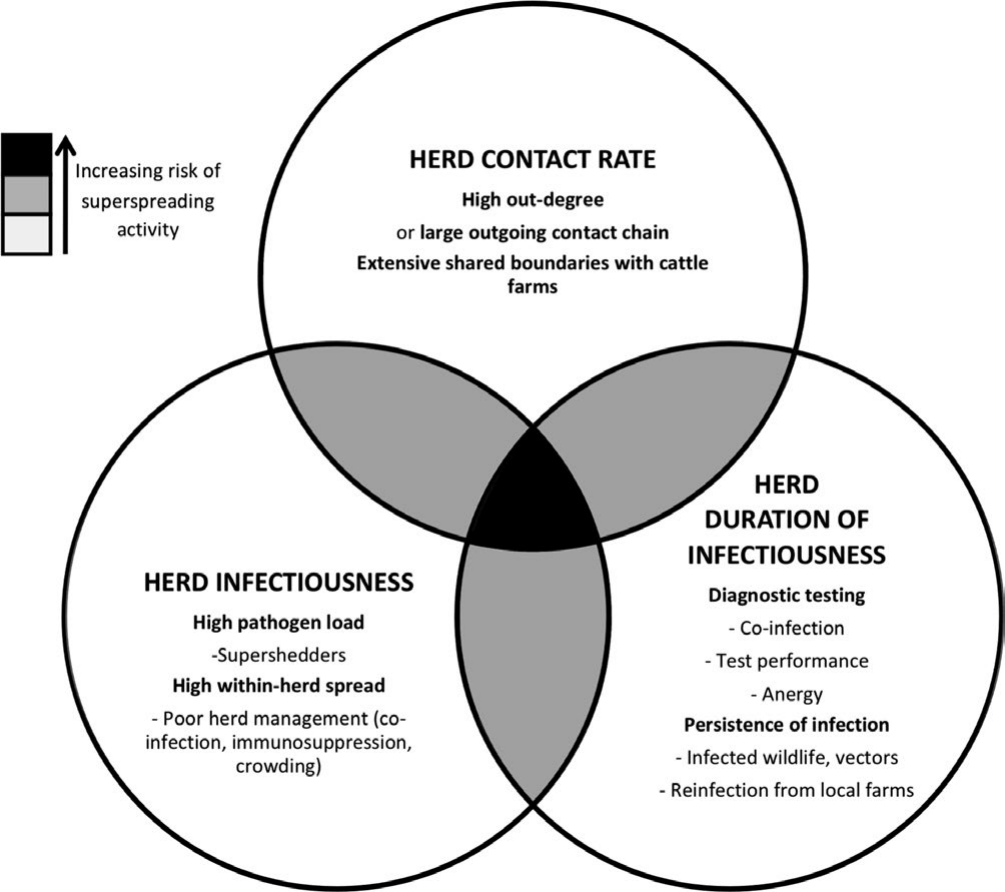
What makes a superspreader farm? Venn diagram showing factors involved in extreme components of R_f_ and the increasing risk of superspreading activity when these factors are combined. These factors are not mutually exclusive and interactions occur between these components, for example the product of infectiousness and contact rate are typically combined to describe the ‘transmission rate’ and factors affecting both infectiousness and the duration of infectiousness such as immunity and co‐infection are common

The transmission rate of an infection is governed by three components: contact rate, infectiousness, and duration of infectiousness (Figure 1) (VanderWaal & Ezenwa, 2016). We use this framework to discuss how a superspreader farm might act. We first consider variation in contact rates among farms that arises from heterogeneity in the scale of cattle movements, both directly and as part of large and complex trading networks. Second, we study how the characteristics of *M. bovis* infection and of individual farms affects the ‘infectiousness’ of farms. Third, we look at factors specific to bTB, such as imperfect diagnostic testing (de la Rua‐Domenech et al., 2006), the general absence of clinical symptoms in infected cattle in Great Britain (Neill, Bryson, & Pollock, 2001), and environmental sources of infection, including wildlife, and how these might contribute disproportionately to the prolonged duration of bTB infection on some farms. Finally, we discuss appropriate control options for putative superspreader farms, that might contribute to bTB control in Great Britain.

## 2 HERD CONTACT RATE

The buying and selling of cattle and their movements among farms are the most obvious and comprehensively‐recorded interactions among herds, and constitute the major potential mechanism for pathogen transmission (Brooks Pollock et al., 2014; Frössling, Ohlson, Björkman, Håkansson, & Nöremark, 2012; Gates, 2014; Palisson, Courcoul, & Durand, 2016). Heterogeneity in trade movements (VanderWaal et al., 2015) is likely to be a key source of variation in R_f_ (Woolhouse et al., 2005)_,_ and quantification of contact rates has been effective in identifying potential superspreaders of infection. Multiple analyses of farm trading networks have found a power‐law distribution (Clauset, Rohilla Shalizi, Newman, & M.E., 2009) for in‐degree (number of farms from which animals are purchased), out‐degree (number of farms to which they are sold), and overall degree (in‐ plus out‐degree) in livestock movement networks, where most premises have few contacts while a few premises have many (Dutta, Ezanno, & Vergu, 2014; Mweu, Fournié, Halasa, Toft, & Nielsen, 2013; Rautureau, Dufour, & Durand, 2011). These directed centrality measures can be used as proxies for a farm's ability to acquire (in‐degree) and transmit (out‐degree) infection (Dubé, Ribble, Kelton, & McNab, 2009). For example, the in‐degree of a farm was found to relate to herd seroprevalence of bovine coronavirus in Sweden (Frössling et al., 2012), and bTB in East Africa (Sintayehu, Prins, Heitkönig, & de Boer, 2017). Second‐order connections (the contacts of contacts) can influence the role an individual might play in disease spread. For instance, in human sexual contact networks, the risk of *acquiring* human immunodeficiency virus was better predicted by the behaviours of partners‐of‐partners than by first order partners alone, and consequently was better for estimating *onward* transmission (Ghani & Garnett, 2000). Eigenvector centrality, a measure that considers both direct and second order contacts, was helpful in determining how influential a farm might be in the spread or maintenance of a theoretical, highly infectious epidemic in Italian cattle movement networks (Natale et al., 2009). In a study of FMD outbreaks, the reproduction number of second order contacts provided good predictions of epidemic size and, when combined with R_f_, provided good estimation of heterogeneities in the dynamics of UK FMD outbreaks (Tildesley & Keeling, 2009). Furthermore, the combining of global clustering and centrality metrics of simulated epidemics with measures of node centrality performed well in detecting superspreading nodes (Fu, Huang, & Sun, 2015).

These measures of centrality, however, do not consider the temporal sequence of events and the analysis of dynamic networks is less well‐developed as it is methodologically and computationally more complex. In some cases, such dynamics have been crucial to our understanding of how pathogens might be transmitted through a network (Enright & Kao, 2018), allowing for the possibility of changing temporal windows for transmission between nodes. The calculation of time‐ordered paths or ‘infection chains’ is one such technique that respects temporal order and gives an indication of the influence of individual nodes. The ingoing infection or ‘ingoing contact chain’ is the network of farms connected to a farm as a result of movements onto that farm (Nöremark, Håkansson, Lewerin, Lindberg, & Jonsson, 2011), and represents the possible sources that may have contributed to acquiring infection during a specified period. Typically these chains are positively skewed with many farms having small contact chains but some having very extensive chains (Fielding, McKinley, Silk, Delahay, & McDonald, 2019; Nöremark et al., 2011), similar to the pattern found for direct contacts in static networks. These very extensive chains of farms aggregated over a period of five years have been associated with an increased risk of *M. bovis* infection in French cattle herds (Palisson et al., 2016), showing that chain magnitude may be useful in predicting which farms might be more likely to spread chronic infections. British cattle herds with more high‐risk farms in their contact chains aggregated over a three year period were associated with increased odds of bTB incidents (Fielding, McKinley, Delahay, Silk, & McDonald, 2020). In choosing the time period over which to study the network, the independent timescales of the movement network and the pathogen should be considered (Kao, Green, Johnson, & Kiss, 2007). If bTB spreads slowly in comparison to a quickly evolving, dynamic network, then the relevant contact networks may become so saturated that they approximate homogenous mixing (Enright & Kao, 2018), making it more difficult to identify potential superspreader farms. However, analytical advances may improve our ability to interrogate such large networks. The use of individual‐based modelling on dynamic network models (Silk et al., 2017) and the application of whole genome sequencing (Trewby et al., 2016) might facilitate further characterization of these transmission pathways.

For bTB, significant data are available on cattle testing and movements, however analysis is constrained by unknown pathogen characteristics such as incubation, infectious and latent periods. To fit these models to available data therefore requires the use of statistical methods that can deal with the presence of hidden states and/or incomplete data. The Bayesian framework allows for models to be fitted which ‘average over’ the missing information. Techniques such as data‐augmented/reversible‐jump Markov chain Monte Carlo (MCMC) (Jewell, Kypraios, Neal, & Roberts, 2009) treat the hidden states as latent variables, which are estimated and then marginalized to generate information on parameters of interest. These approaches are highly computationally complex and challenging to code for large‐scale diseases on networks. Alternative simulation‐based approaches, such as particle MCMC and Approximate Bayesian Computation (Kosmala et al., 2016; McKinley, Cook, & Deardon, 2009) may be more tractable, albeit often at the cost of introducing approximations. A popular frequentist approach is the maximum likelihood via iterated filtering method (Ionides, Bretó, & King, 2006), and there is much ongoing research aimed at tackling these modelling challenges.

## 3 HERD INFECTIOUSNESS

While the number of movements and trading partners will undoubtedly be a principal driver to increase the influence of certain premises, other factors govern whether those movements result in transmission, i.e. herd/farm infectiousness. For highly transmissible infections with high within‐herd prevalence, it is likely that any movement would transmit infection, regardless of the farm characteristics. However, where the disease spreads slowly within a herd, as is typically the case with bTB, the risk of selling an infected animal is more variable and farm factors are likely to be more influential. We discuss such factors, including disease prevalence, herd immunity, presence of multiple infections and the type of animals being sold, all of which might influence the likelihood of selling infected animals.

Supershedders, highly infectious individuals releasing more infectious agents than others in their group (Chase‐Topping, Gally, Low, Matthews, & Woolhouse, 2008), can increase herd prevalence. Their presence in the herd reduces the efficacy of whole‐herd control measures, due to heterogeneity generated in transmission rates (Lanzas et al., 2008). Supershedding can be driven by host genotype, behaviour, signalment (age, sex and breed) (Craft, 2015), co‐infection (Lass et al., 2013), immunosuppression (Stein, 2011), enhanced susceptibility, or strain pathogenicity of the infecting agent (Matthews et al., 2009). Whilst host genotype has a well‐established link to resistance against particular pathogens (Tsairidou et al., 2014), direct links have now been identified between host genotype and infectiousness (Anacleto et al., 2019). This may be crucially important in less genetically‐diverse livestock populations, where the impact of more‐infectious host genotypes could be amplified in herds dominated by a particular sire or pedigree line. Heterogeneity in bacterial shedding has been observed in cattle infected with *Escherichia coli* O157 (Chase‐Topping et al., 2008), *Salmonella enterica* (Lanzas et al., 2008) and *Mycobacterium avium* subspecies *paratuberculosis* (MAP), the causative agent of Johne's disease in cattle (Pradhan et al., 2011). Supershedders of MAP have been suggested to cause herd‐mates to passively shed the bacteria, while remaining tissue culture negative (Pradhan et al., 2011). Stress from movements and from weaning have been implicated as risk factors for supershedding of *E. coli* O157 (Chase‐Topping et al., 2007). Supershedders of *M. bovis* have been identified in red deer (*Cervus elaphus*) and badgers (Santos, Almeida, Gortázar, & Correia‐Neves, 2015; Wilkinson et al., 2000). In cattle, the most likely route to being a supershedder of *M. bovis*, i.e. an animal excreting more pathogen than others, would be one with undetected, late‐stage infection (Houlihan, Dixon, & Page, 2008), although it is not known whether this state is linked to host genotype.

The risk of movements transmitting infection depends on the the type of animal being traded (Gates, Humphry, Gunn, & Woolhouse, 2014). Trading male or female breeding cattle might present a relatively greater risk of transmission for various infections, including *M. bovis* (Chase‐Topping et al., 2007; Gates et al., 2014; Griffin et al., 1993), which is likely to be associated with the more advanced age of breeding animals compared to fattening stock, and the increased risk of infection with age (Brooks Pollock et al., 2013; Humblet, Boschiroli, & Saegerman, 2009). Dairy cattle may be more likely to react to PPD‐tuberculin tests such as the SICCT, and therefore present a lower risk if they have been recently tested (Downs, Broughan, Goodchild, Upton, & Durr, 2016).

At times, herd composition and farm practices may interact to drive variation in herd infectiousness. Beef herds rearing animals to sell directly to slaughter (finishing herds) do not pose an onward disease transmission risk, though it is commonplace in Great Britain for animals to be sold as ‘stores’ (animals reared for beef but not ready for slaughter) and traded among multiple fattening herds (Robinson & Christley, 2007). This type of herd tends to house cattle from different sources, usually purchased via markets (Robinson & Christley, 2007). Mixing of cattle from many source farms can have physiological effects that may increase susceptibility to infections (Proudfoot, Weary, & von Keyserlingk, 2012). Combined with potential exposure to pathogens from several farms, this can facilitate within‐herd transmission of multiple infections (Griffin, Chengappa, Kuszak, & McVey, 2010). As co‐infection can alter host immune responses and increase pathogen shedding (Lanzas et al., 2008), it may therefore increase herd infectiousness. Vaccination, diagnostic testing, good management and sourcing of animals from fewer, disease‐free farms may all reduce this risk, but for diseases such as bTB where vaccination is not available for cattle and where tests have low sensitivity, these risks are more difficult to manage.

A final farm‐level characteristic that will influence both herd infectiousness and its duration is husbandry, as poor hygiene can increase exposure to infection and hence increase herd infectiousness. The physical environment (poor hygiene, exposure to multiple pathogens, injury) has been shown to have a direct impact on the individual, which can increase the risk of disease (Proudfoot et al., 2012). They also note the indirect impact of social stressors (overcrowding, mixing of groups, isolation) on host physiology (immunomodulation, low resilience, chronic inflammation), which can then increase risks of infection and disease progression. Winter housing of cattle can cause social stress due to mixing of groups and crowding and, where there is poor ventilation, transmission of airborne pathogens can increase (Gorden & Plummer, 2010). Hence, increased seroconversion of dairy cattle to bovine herpesvirus type‐1 has been associated with winter housing (Woodbine et al., 2009) and there is some evidence for housing as a risk factor for bTB transmission (Vial, Miguel, Johnston, Mitchell, & Donnelly, 2015). Although most bTB incidents now have very few reactors (Animal & Plant Health Agency, 2017), suggesting low within‐herd transmission, the sharing of a confined, poorly ventilated environment by supershedders or many high‐risk cattle and susceptibles may be sufficient to cause a superspreading event within a farm (Lloyd‐Smith et al., 2005), and thus increase herd infectiousness.

## 4 DURATION OF INFECTIOUSNESS

Prolonged infectiousness of a herd, through misdiagnosis (of novel or rare infections), undetected infections (if asymptomatic) (Drosten, Lau, Preiser, So, & Yam, 2003) or poor test sensitivity, can facilitate the spread of disease. The duration of the infectious period can be altered by treatment, vaccination or culling (Thurmond, 2003). Decisions on whether and how to apply these control methods are generally based on results of diagnostic testing. In the case of bTB infection in Great Britain, most infected cattle do not present with clinical signs and the principal statutory SICCT test for *M. bovis* has low sensitivity, that can be markedly lower in certain circumstances, allowing some herds to have an extended infectious period, enabling them to act as superspreaders over time.

Co‐infection may alter the host response to infection and host infectiousness (Lass et al., 2013), which can thereby alter the non‐specific immune responses measured by diagnostic tests. In cattle co‐infected with liver fluke (*Fasciola hepatica*) and *M. bovis*, shifts in immunity from T‐helper cell 1 to T‐helper cell 2 responses have been implicated in reducing the immune response to the tuberculins used in SICCT testing in England and Wales (Claridge et al., 2012), though studies using data from Northern Ireland found no such relationship at individual or herd‐level (Byrne, Graham, et al., 2017).

*Mycobacterium avium* subsp. *paratuberculosis* (MAP) is the causative agent of Johne's disease, a chronic enteric infection that is estimated to affect about a third of UK cattle herds (Animal & Plant Health Agency, 2015; Barratt et al., 2018). Co‐infection with bacteria in the *M. avium* complex has been reported to reduce the sensitivity of SICCT and gamma interferon (IFN‐γ) testing for *M. bovis* in small‐scale studies, through cross‐reaction of antigens and an increase in response to the avian tuberculin of the SICCT test (Álvarez et al., 2009; Hope et al., 2005). Large‐scale epidemiological studies in Northern Ireland have found visible *M. bovis* lesions that were more likely to be observed in cattle positive to both *M. bovis* and *M. avium* tuberculins compared to those with only an *M. bovis* reaction. If a positive response to *M. avium* tuberculin can be used as a proxy for potential MAP infection, this could indicate that MAP infection delays the detection of *M. bovis* infections (Byrne, Graham, et al., 2017). Furthermore, Northern Irish herds experiencing a bTB incident were more likely to be sero‐positive for MAP (Byrne, Graham, Milne, Guelbenzu‐Gonzalo, & Strain, 2019) and questionnaire data suggested that dairy farms in England and Wales that had experienced MAP infection in the preceding 12 months were 4.7 times more likely to have a bTB incident (Broughan et al., 2016). Given the likely high prevalence of MAP in UK herds, further investigation into this relationship is warranted.

Bovine viral diarrhoea virus (BVDV) is widespread in England and Wales (Charleston, Hope, Carr, & Howard, 2001) and immunosuppression in acute viral infection leaves animals susceptible to concurrent infections. Animals infected with *M. bovis* and acute BVDV infection showed suppression of IFN‐γ production when stimulated with tuberculin (Charleston et al., 2001), which was associated with a particularly severe outbreak of bTB in a group of calves (Monies, 2000). However, recent studies in Northern Ireland have found no positive association between BVDV and bTB infection at animal or herd levels (Byrne, Graham, et al., 2017; Byrne, Guelbenzu‐Gonzalo, et al., 2017). In summary, co‐infection with certain pathogens can lead to changes in the performance of diagnostic tests, reduce the detection or confirmation of infection and may leave hidden infection in the herd. Herds with high prevalences of these diseases have a greater chance of prolonged infectiousness, increasing their risk of becoming superspreader farms.

Immune responses change during disease progression and during the host's lifetime, and diagnostic tests therefore vary in their performance, depending on when the host is tested (Schukken et al., 2015). For some infections, longitudinal testing is required to increase test performance where single test results are not sufficiently robust (Schukken et al., 2015). As *M. bovis* infection in cattle progresses, the initial cell‐mediated immune response wanes and some infected animals can become unresponsive or ‘anergic’ to SICCT testing (Neill et al., 2001). Undetected by routine tests, these animals remain in the herd, and over time may develop lesions and the capacity to disseminate infection, potentially acting as supershedders (Houlihan et al., 2008). Annual SICCT testing in areas of higher bTB risk means that cattle should be detected before the natural cell‐mediated response wanes, so that the number of naturallyanergic cattle is likely to be low. However, their potential to persist undetected in an infectious state may be epidemiologically significant. In addition to the natural progression of infection, the sensitivity of the test may decrease over time, due to repeated exposure to tuberculin (Coad et al., 2010), for instance, in prolonged bTB incidents where herds are SICCT tested at 60 day intervals. Temporary anergy to the SICCT test can also develop in periods of stress, around parturition (Li, 2016), and when corticosteroids are administered (Phillips, Foster, Morris, & Teverson, 2002), allowing evasion of diagnosis if testing is performed at this time. Some animals that are not fully anergic may exhibit a partial cell‐mediated response to the SICCT test, and present as inconclusive reactors (IRs). These animals are tested 60 days later and if they test negative (as one would expect of temporarily anergic animals) can remain in the herd. However, IRs that retest negative after 60 days and remain in the herd have 12 times greater risk of testing positive at the next routine SICCT test or at slaughter (Clegg et al., 2011), suggesting that they are false negatives at retesting, perhaps due to co‐infection, anergy or test sensitivity, presenting a prolonged source of transmission within the herd. Since 2017 in England, IRs have not been allowed to leave the farm, in an attempt to reduce risks of onward transmission between‐herds (Department for Environment, Food & Rural Affairs, 2017), although the risk of within‐herd transmission remains. Prior to 2018, the SICCT test and the IFN‐γ test were the only *ante‐mortem* tests approved to diagnose bTB in British cattle, both detecting cell‐mediated immunity, however, policy now allows the exceptional use of a non‐validated test, which detects antibodies, if they are present in these anergic animals (Animal & Plant Health Agency, 2018).

Local contacts between neighbouring cattle or wildlife is considered an important factor in bTB epidemiology (Brooks Pollock et al., 2014). Spatial clustering analysis of bTB data from England in 2005 showed only weak evidence for clustering of disease on a county level (Green & Cornell, 2005). However, herd‐level risk factor studies have found that risks of bTB are greater for farms whose neighbours have a history of infection (Fielding et al., 2020; Skuce, Allen, & McDowell, 2012). A study of *M. bovis* transmission in France, where infection is rare, combined the cattle movement network with a ‘spatial neighbourhood’ based on geographic proximity of farms (Palisson et al., 2016). They estimated that 73% of infection (the population attributable fraction) could be removed if local transmission was eliminated. Brennan, Kemp, and Christley (2008) studied the contacts of cattle farms in North‐West England in respect to contractors and companies, shared equipment and employees. They found that frequency of such contacts exhibited the same heterogeneity as in animal movement networks. *M. bovis* can survive in infected cattle faeces in slurry for up to 6 months (Scanlon & Quinn, 2000) and on pasture for 1–6 months (Williams & Hoy, 1930), thus the application of slurry from infected farms or shared contractors and equipment may present a risk of bTB persistence on infected farms or a source of infection for uninfected farms. Badgers infected with *M. bovis* present a potential source of infection for cattle, most likely via indirect contact at latrines and contamination of pasture and feed (Drewe et al., 2013; Silk et al., 2018; Woodroffe et al., 2016). The persistence of infection in local badgers may therefore facilitate persistence in cattle, particularly where transmission occurs in both directions, creating a cycle of reinfection.

Undoubtedly, contiguous spread of *M. bovis* contributes to persistence and repeat infections within cattle herds. However, to be defined as a superspreader a host or herd must be responsible for seeding a disproportionately high number of secondary infections. When considering individual farms as superspreaders of infection, local spread is likely to be largely constrained by farm boundaries, and limited by the number of neighbouring farms. Therefore, we consider the role of contiguous transmission in superspreader farms primarily as a factor that might increase the risk of a farm becoming infected, but then would need to additionally combine with a high contact rate, high infectiousness or long duration of infection, to result in a disproportionate number of secondary infections.

## 5 IDENTIFICATION OF SUPERSPREADER FARMS ‐ WHERE SHOULD WE TARGET EFFORT FOR EFFICIENT AND EFFECTIVE CONTROLS?

In addition to exhibiting extreme values for at least one component of R_f_, a superspreader must clearly be infected and have some contact with other hosts. For example, if infection is removed from a farm with a high contact rate, or if a highly infectious farm no longer contacts other farms (e.g. movement restrictions are effectively applied), then they can no longer function as superspreaders. Nevertheless, they might still be considered to retain superspreading potential and so it might be prudent to target such farms for additional surveillance. The three components of R_f_ can combine to increase the risk of superspreading occuring (Figure 1), and therefore increase the impact of an individual farm on disease dynamics. The identification of superspreaders provides an opportunity to focus or intensify control measures such as treatments, vaccination, isolation, restrictions, to gain disproportionate benefits. Lloyd‐Smith et al. (2005) calculated that where half of all control effort is focused on the 20% of population responsible for the majority of disease transmission, it is up to three times more effective than random control. Therefore, in this section, we look at which existing control measures and which novel approaches might be used to target superspreader farms.

Livestock markets and some farm premises have a high throughput of animals, termed ‘hubs’ in networks analysis (Robinson & Christley, 2007), and are especially important in governing the size of epidemics of highly transmissible infections. Emergency disease control measures targeted at these hubs have been effective in limiting epidemic size in FMD and HPAI outbreaks (Green, Kiss, & Kao, 2006; Molia et al., 2016). Büttner, Krieter, Traulsen, & Traulsen (2013) found that to achieve a 75% reduction in estimated epidemic size in pig movement networks, removing farms based on high out‐degree or large outgoing contact chains allowed the fewest farms to be removed from the network (1.4% and 1.5%, respectively). Models based on disease transmission through cattle movement networks have shown that removal of the 20% of farms contributing most to R_0_ resulted in a 97%–99% reduction of R_0_ (Volkova, Howey, Savill, & Woolhouse, 2010). These studies demonstrate that focusing efforts on these small but highly influential groups can be more effective than population‐wide control in reducing estimates of final epidemic size. However, it is useful to note that the effectiveness of targeted control measures is likely to be proportional to the extent of heterogeneity in that population, such that the more influential the ‘top’ nodes, the more benefit that can be gained from their removal. Where populations exhibit less heterogeneity, more nodes or farms must be targeted to achieve similar levels of risk reduction (Brown, Marshall, Mitchell, & Byrne, 2019), thus the merits of this approach should be assessed for each population.

Ideally livestock movement restrictions should facilitate a reduction in high‐risk trading for an appropriate time period, whilst maintaining the ability to move low‐risk stock on and off the farm where necessary. More bespoke restrictions between trading partners are discussed by Gates and Woolhouse (2015), where farms with high in‐degree (mixing of animals from multiple sources) are prevented from selling to farms with high out‐degree, thereby reducing the risks associated with this behaviour. These trading restrictions decrease the likelihood of high‐risk trade paths reconfiguring and allow the farm other outlets for trade to sustain their business. If farms are completely removed from a network (Enright & Meeks, 2015), the remaining farms that were connected to them tend to find new partners with which to trade (Brouwer, Bartels, Stegeman, & van Schaik, 2012). However, these may have a similar risk of disease transmission as the original partners, creating a new structure as risky as the previous one (Brouwer et al., 2012). For bTB control, movement restrictions are placed on the individual farm when a reactor is found, and are later lifted following two consecutive clear SICCT tests. However, undetected infection on the farm (Conlan et al., 2012) may mean that these restrictions are lifted prematurely. Extended movement restrictions on those farms with recurrent bTB incidents, high out‐degree or an exceptionally large outgoing contact chain, might provide an effective, risk‐based addition to current measures. Limiting sales from high‐risk farms to only approved finishing units or direct to slaughterhouses (as is currently allowed for some farms under bTB restrictions) (Animal & Plant Health Agency, 2014) may be effective in limiting the spread of infection.

To discourage trading from higher to lower‐risk farms, herds can be allocated a risk score. Farms in New Zealand have a designated bTB status score from 1 to 10, denoted by the number of years since the last bTB incident, defaulting to 10 if they have never had a bTB incident. Their score is, however, superceded by the lowest score of a farm with which they trade (Enticott, 2014), thereby encouraging farmers to trade with ‘less risky’ farms. In Britain, Adkin et al. (2015) developed a scoring system based on the previous bTB history of farms, movements from higher risk areas, local bTB prevalence and herd size, to inform risk‐based purchasing and enable farmers to make informed decisions. Although this risk‐score has not yet been used in practice to any significant extent, the Cattle Health Certification Standards (CHeCS) scheme have launched a voluntary bTB accreditation‐scheme based similarly on years free of bTB incidents, but not influenced by trading history. These schemes attempt to lower the risks associated with purchasing cattle, while allowing farm business to continue. In 2012, a survey of cattle farmers in low and high risk areas of England found that 53% of respondents said they would find such a scheme valuable (Little, Wheeler, & Edge, 2017). Inevitably, the success of such voluntary initaitives is dependent on uptake, which to date has been very low in Britain.

Network analysis of between‐farm movements allows us to detect herds with the potential to act as superspreaders. An exciting area of future work is combining these findings with whole genome sequencing of the pathogen, which can provide uniquely fine‐scale insights into transmission pathways. This might allow researchers to more easily identify superspreading events and to disentangle the relative contributions of cattle to cattle transmission of *M. bovis* and the role of wildlife in maintaining infection on certain farms (Crispell et al., 2019; Kao, Price‐Carter, & Robbe‐Austerman, 2016). This type of forensic‐level analysis can only be applied in farms where infections have been detected, but might be effectively combined with increased testing on farms with high potential to act as superspreaders.

The identification of genetic resistance to bTB has led to the development of genetic indices that can be used to help breed for reduced susceptibility (Tsairidou et al., 2014), and modelling suggests that additionally selecting for genotypes associated with lower infectiousness would further accelerate the effectiveness of this approach (Tsairidou, Anacleto, Woolliams, & Doeschl‐Wilson, 2019). However, even if a genetic marker for infectiousness can be found (Tsairidou et al., 2018), these approaches may still take several generations to achieve substantial results (Tsairidou et al., 2019). In the short‐term, if farms with high proportions of more‐infective genotypes can be identified, we might proactively apply more‐sensitive or more frequent testing regimes to reduce the potential for within‐herd or onward spread of infection.

Vaccination can be effective in limiting spread within a homogenous population. However, as heterogeneity of R_i_ increases, vaccine efficacy must be higher to achieve the same level of control (Lanzas et al., 2008). Therefore, preferential targeting of potential superspreader farms for vaccination might be better directed towards those farms which ‘superspread’ via higher contact rates rather than higher infectiousness. In models where 80% of individuals with high contact rates were vaccinated against influenza, there was a 91% disease reduction predicted for the whole population (Weycker et al., 2005). The use of Bacillus Calmette‐Guerin (BCG) vaccination for control of bTB in cattle is currently prohibited under EU law (European Economic Community, 1977). Variable efficacies have been reported for the use of BCG vaccine in cattle (Waters, Palmer, Buddle, & Vordermeier, 2012) and its use in combination with the existing test and slaughter policy requires a test that differentiates infected from vaccinated animals (DIVA) (Conlan et al., 2015). Although DIVA testing is being developed (Swift, Convery, & Rees, 2016), there are concerns regarding low specificity (Conlan et al., 2015), economic viability, and the practicalities and regulation of field trials to prove reduction in transmission (Conlan, Vordermeier, de Jong, & Wood, 2018). The use of vaccination against *M. bovis* in UK cattle remains speculative, but if it became available then targeting potential superspreader farms might represent an effective first step in reducing disease spread.

## 6 CONCLUSION

There are mechanisms within current farm practices and bTB controls that could facilitate the existence of superspreader farms for bTB. Farms with influential roles in cattle movement networks are able to sell animals to many different premises. The risk of these animals being infected might be increased by high within‐herd spread. The opportunity to sell infected animals increases with the duration of infection on the farm and this can be increased by factors that impair the sensitivity of routine testing and ongoing reinfection from within the herd, or local sources. In a minority of cases, we expect that a high contact rate might be combined with high infectiousness to create a superspreader farm. The challenge is then to identify these disproportionately important farms in ‘real time’. Where the three aspects of superspreading (contact rate, infectiousness and duration of infectiousness) vary over time, superspreading may be a transient phenomenon. It is important that any restrictions placed on these farms occur only while they are at a high risk of transmitting disease and, ideally, in a way that minimizes impacts on farm businesses.

It is probable that the operators of farms that might exhibit superspreader characteristics are unaware of their potential impact on bTB disease dynamics. Further research may reveal whether identification of these farms can encourage such farmers to adapt their practices and mitigate potential risks for the wider benefit of the industry. The categorization of some farms into this higher‐risk bracket is intended to create an opportunity for greater resources to be directed at these farms, rather than remove them from the network completely, as previous studies have shown this is likely to be ineffective in the long term (Brouwer, Bartels, Stegeman, & van Schaik, 2012).

Surveillance is crucial to controlling infection within and between‐herds. Targeting existing surveillance efforts at farms with the potential to become superspreaders, by means of their high connectivity, could therefore potentially reduce their impact. Frӧssling, Nusinovici, Nöremark, Widgren, and Lindberg (2014) found that using in‐degree and ingoing infection chains to target surveillance detected more positives than random approaches. For bTB, it is crucial to improve the detection of infected animals on potential superspreader farms. Increasing the frequency of routine testing on high‐risk superspreader herds, reduces the time to detection and removal of animals, and so can reduce the duration and intensity of infectiousness at the farm scale. The sensitivity of routine testing can also be increased by using the severe interpretation of the SICCT test (decreasing the cut‐off criteria which define reactors), a non‐comparative Single Intradermal Cervical Test (de la Rua‐Domenech et al., 2006), or the IFN‐γ test. However, increased sensitivity is typically associated with a loss of specificity, which if applied across a very large population would lead to unacceptably high numbers of false positive reactors being culled and the unnecessary imposition of restrictions on farm businesses. The IFN‐γ test is used in addition to the SICCT test when specific criteria are met and can be particularly useful in detecting bTB‐positive animals that have become desensitised to the SICCT test as a result of repeated testing (Coad et al., 2010), those co‐infected with other *Mycobacterium* spp., and animals in early stages of infection (de la Rua‐Domenech et al., 2006). Use of more specific antigens such as ESAT‐6/CFP10 in the IFN‐γ test may offer additional diagnostic power in herds with animals vaccinated with BCG (van Pinxteren, Ravn, Agger, Pollock, & Andersen, 2000) and/or known co‐infection with other mycobacteria (Aagaard et al., 2010). The aim of identifying potential superspreader farms for bTB control is to better understand the mechanisms by which they might operate and to adapt disease management approaches to exploit their influential role in disease transmission, thereby enhancing control of this costly endemic infection. Targeted use of more sensitive and less specific measures or combined testing protocols, on potential superspreader farms, would minimize impacts on the wider industry whilst maximizing disease control benefits.

## CONFLICT OF INTEREST

The authors declare no conflicts of interest.

## AUTHOR CONTRIBUTION

**Helen R. Fielding:** Conceptualization; Formal analysis; Investigation; Writing‐original draft; Writing‐review & editing. **Trevelyan J. McKinley:** Investigation; Supervision; Writing‐review & editing. **Richard J. Delahay:** Funding acquisition; Investigation; Supervision; Writing‐review & editing. **Matthew J. Silk:** Investigation; Writing‐review & editing. **Robbie A McDonald:** Conceptualization; Funding acquisition; Investigation; Project administration; Supervision; Writing‐review & editing.

## ETHICS STATEMENT

The authors confirm that the ethical policies of the journal, as noted on the journal's author guidelines page, have been adhered to. No ethical approval was required as this is a review article with no original research data.

### PEER REVIEW

The peer review history for this article is available at https://publons.com/publon/10.1002/vms3.358.
